# Vestibular Perceptual Thresholds Increase above the Age of 40

**DOI:** 10.3389/fneur.2016.00162

**Published:** 2016-10-03

**Authors:** María Carolina Bermúdez Rey, Torin K. Clark, Wei Wang, Tania Leeder, Yong Bian, Daniel M. Merfeld

**Affiliations:** ^1^Harvard Medical School, Boston, MA, USA; ^2^Jenks Vestibular Physiology Laboratory, MEEI, Boston, MA, USA; ^3^University of Colorado at Boulder, Boulder, CO, USA; ^4^Department of Medicine, Brigham and Women’s Hospital, Boston, MA, USA

**Keywords:** vestibular, perception, thresholds, aging

## Abstract

We measured vestibular perceptual thresholds in 105 healthy humans (54F/51M) ranging from 18 to 80 years of age. Direction-recognition thresholds were measured using standard methods. The motion consisted of single cycles of sinusoidal acceleration at 0.2 Hz for roll tilt and 1.0 Hz for yaw rotation about an earth-vertical axis, inter-aural earth-horizontal translation (*y*-translation), inferior–superior earth-vertical translation (*z*-translation), and roll tilt. A large subset of this population (99 of 105) also performed a modified Romberg test of standing balance. Despite the relatively large population (54F/51M), we found no difference between thresholds of male and female subjects. After pooling across sex, we found that thresholds increased above the age of 40 for all five motion directions investigated. The data were best modeled by a two-segment age model that yielded a constant baseline below an age cutoff of about 40 and a threshold increase above the age cutoff. For all subjects who passed all conditions of the balance test, the baseline thresholds were 0.97°/s for yaw rotation, 0.66°/s for 1-Hz roll tilt, 0.35°/s for 0.2-Hz roll tilt, 0.58 cm/s for *y*-translation, and 1.24 cm/s for *z*-translation. As a percentage of the baseline, the fitted slopes (indicating the threshold increase each decade above the age cutoff) were 83% for *z*-translation, 56% for 1-Hz roll tilt, 46% for *y*-translation, 32% for 0.2-Hz roll tilt, and 15% for yaw rotation. Even taking age and other factors into consideration, we found a significant correlation of balance test failures with increasing roll-tilt thresholds.

## Introduction

Data suggest that, on average, females and males have a significantly different number of vestibular afferent fibers (1) and that a significant difference in the size of the vestibular labyrinth exists (2). Such anatomical differences could contribute to behavioral differences, but studies utilizing standard clinical vestibular assays (3–8) have found no significant sex effects. Nonetheless, differences could exist. Wall and colleagues reported a very small, but significant, difference in the VOR phase at 0.005 Hz in a population of 25 males and 25 females (9). Benson reported perceptual translation thresholds (i.e., the smallest motion that can be reliably perceived as leftward or rightward) for females that were roughly 40% lower than for males for each of the three translation directions (7), but this difference was not statistically significant. Similarly, yaw rotation thresholds were reported to be about 20% lower in females than males (8), but again, this difference was not statistically significant. Given these data, we felt that a sex effect deserved study using a larger sample.

We chose to measure vestibular thresholds for a variety of reasons. (1) Threshold testing uses small motions that typically are well tolerated. (2) Like other threshold measures (e.g., auditory thresholds), vestibular thresholds have direct functional relevance. (3) Thresholds have been shown to be a sensitive measure of vestibular function that has been shown to identify specific peripheral vestibular deficits (10). (4) Thresholds have shown great promise to help diagnose central disorders such as vestibular migraine (11, 12), which may be the most prevalent vestibular disorder. (5) Unlike other vestibular responses such as the VOR, one previous study was unable to demonstrate adaptive perceptual threshold changes even following substantial training efforts (13) – possibly because the brain receives little information to drive adaptation during threshold-level motion. (6) Thresholds can provide a comprehensive assay of many aspects of vestibular function – including perception, all peripheral end organ pairs, central vestibular functions, etc. – that are straightforward to interpret and can be compared across motion types (i.e., translation, tilt, and rotation) relative to normal.

Earlier vestibular threshold studies have come to different conclusions regarding the effect of age on rotation thresholds and translation thresholds. One study (14) measured thresholds for yaw rotation in a group of 19 younger subjects, aged 20–26, and a group of 16 older subjects, aged 63–84, and found no significant effect of age. Similarly, Seemungal and colleagues (15) reported no difference in yaw rotation thresholds between a group of 14 young (mean age of 23) and 9 older (mean age of 63) normal subjects. Each of these reports is consistent with another study (16) of 24 normal subjects between the ages of 21 and 60 that found no effect of age on yaw rotation thresholds.

While published studies do not show a significant correlation of yaw rotation thresholds with age, there is evidence to suggest that translational thresholds do correlate with age. However, one of the studies that did not find a correlation of yaw rotation threshold with age (16) did report a correlation with age for thresholds measured using naso-occipital (*x*-axis) and inter-aural (*y*-axis) translations. Furthermore, Agrawal and colleagues (17) reported that thresholds of 42 normal subjects demonstrated a significant positive correlation with age for naso-occipital (*x*-axis) and inferior–superior (*z*-axis) translation but not for inter-aural (*y*-axis) translation, and another recent paper (18) reported that translation thresholds for 42 normal subjects were significantly correlated with age for naso-occipital (*x*-axis), inferior–superior (*z*-axis), and inter-aural (*y*-axis) translations. Finally, Kingma (19) reported that for a population of 28 healthy subjects between the ages of 22 and 60 (7 subjects/decade), thresholds increased linearly with age for naso-occipital (*x*-axis) translation but found no correlation for inter-aural (*y*-axis) translation thresholds.

Before proceeding, we also note that non-vestibular cues (e.g., somatosensory and proprioceptive) may contribute to these thresholds, but a previous study showed bilateral vestibular defective patients have significantly higher thresholds (20), suggesting a predominant influence of the vestibular cues.

Given the earlier findings, we decided to include a larger number of healthy normal subjects (54 females and 51 males) than reported in previous investigations. We specifically targeted our recruitment to obtain age- and gender-matched subjects for each decade spanning an age range between 18 and 80. We measured direction-recognition thresholds in the dark for (a) yaw rotations – transduced primarily by the lateral semicircular canals, (b) superior–inferior (*z*-axis) translations – transduced primarily by the saccular organs, (c) inter-aural (*y*-axis) translations – transduced primarily by the utricular organs, and (d) roll tilts – transduced primarily by the vertical canals and the utricular organs. We emphasize that this study is the first to look at age effects for roll-tilt thresholds; the importance of this is emphasized by recent reports of lowered thresholds in patients suffering vestibular migraine (11, 12).

## Materials and Methods

Perceptual thresholds were sampled in 105 subjects, 54 females and 51 males, between the ages of 18 and 80. All subjects filled out a general health questionnaire to confirm that they qualified to participate, including the absence of vestibular symptoms. Menstrual cycle status and diagnosis of migraine were determined *via* two separate questionnaires. A standing balance test was used to objectively evaluate balance function. Threshold data collection methods generally mimicked those used by Valko and colleagues (20), but data were collected for only a small subset of the frequencies sampled in that earlier study. Specifically, for each subject, yaw rotations were applied about an earth-vertical axis at 1 Hz, *y*-translations were applied along an earth-horizontal axis at 1 Hz, *z*-translations were applied along the earth-vertical axis at 1 Hz, and roll tilts about a head-centered earth-horizontal axis were applied at 0.2 and 1 Hz. Participation in the study took about 3 h including at least two breaks. Informed consent was obtained from all subjects as dictated by the Declaration of Helsinki, and the study was approved by the MEEI Human Use Committee.

### Questionnaires

A short health questionnaire was administered to all subjects for screening purposes. History of current and previous diseases, with an emphasis in neurological, otologic, vestibular, and chronic uncontrolled diseases, and medications was obtained. Acting conservatively, subjects diagnosed with any major health problem or under medications that could potentially affect vestibular function or decision making were excluded, as were subjects with any history of vestibular symptoms. As just one example, subjects with vestibular migraine would typically have been excluded because of their occasional symptoms.

Women were asked to fill out a separate questionnaire to establish menstrual cycle status (premenopausal, postmenopausal, or other). For premenopausal women, length and regularity of cycles, start of current cycle (i.e., first day of menstrual bleeding), and current use of hormonal contraception was recorded.

Because prevalence of migraine is known to be higher in females (21), we considered migraine as a potential confounding factor for our analyses. The Migraine Screen Questionnaire (MS-Q) developed and validated by Láinez et al. (22, 23) was administered to confirm history of migraine and/or to detect hidden migraine. A MS-Q score ≥4 was considered positive.

### Balance Testing

To assess balance function, the modified Romberg test of standing balance on firm and compliant support surfaces (24) was performed. This balance test consists of four steps. Each step must be passed in order to move to the next step. All steps are performed standing with feet together and arms crossed. To pass the first step, each participant had to stand on the floor for 15 s with eyes open. To pass the second step, they had to stand on the floor for 15 s with eyes closed. To pass the third step, they had to stand on memory foam with eyes open for 30 s. To pass the final step, they had to stand on the foam with eyes closed for 30 s. This final test condition primarily assesses vestibular function (24, 25), since visual contributions are eliminated and the foam makes kinesthetic cues unreliable. The balance test was scored on a pass/fail basis. Failure was defined as participants needing to open their eyes or arms or move their feet to maintain stability before the end of the trial. All subjects were allowed two trials at each step.

### Motion Stimuli and Psychophysical Threshold Tests

The motion paradigms and psychophysical tests employed to measure perceptual thresholds for this study have been previously published in detail (26, 27), so are described briefly herein. Motion stimuli were generated with a Moog 6DOF motion platform. Motion stimuli were single cycles of sinusoidal acceleration (either linear acceleration or angular acceleration) [$a(t)=A\text{sin}(2\text{π}ft)=A\text{sin}\left(\frac{2\text{π}t}{T}\right)$, where *A* is the acceleration amplitude and *f* is the motion frequency]. We present thresholds using the peak velocity of each stimulus. As shown in earlier papers [e.g., Ref. (8, 26)], this yields bell-shaped velocity trajectories having a maximum velocity of *v*_max_ = *A*/(π*f*).

Subjects were seated in an upright position, held *via* an adjustable five-point harness and a helmet. To minimize other sensory cues, motions were performed in the dark in a light-tight room, all skin surfaces except the face and hands were covered, and noise-canceling headphones played constant amplitude white noise during the motions to mask any auditory cues and to indicate the time period when each motion occurred.

A three-down/one-up (3D/1U) adaptive staircase was used to target stimuli near threshold (28, 29). To minimize training effects, suprathreshold practice trials were administered until each subject understood and was comfortable with the task before each set of trials. Each block consisted of 100 trials, where a single motion stimulus was provided per trial. One hundred trials was considered adequate because an earlier study (29) showed that 100 trials yielded methodological threshold variations of just 18% – much less than the intra-subject variations reported previously by Benson (7, 8). Furthermore, 200 trials, while roughly doubling test time, yielded just an incremental improvement in threshold precision (from 18 to 13%). Until the first mistake, the stimulus was halved after three correct responses at each level. From this point onward, the size of the change in stimulus magnitude was determined using parameter estimation by sequential testing (PEST) rules (30). For all conditions, initial stimuli were set at a level that was suprathreshold for the vast majority of subjects. Yaw rotations began at a *v*_max_ = 4°/s, *y*-translations at *v*_max_ = 4 cm/s, *z*-translations at *v*_max_ = 16 cm/s, and roll tilts at *v*_max_ = 3°/s for 1-Hz stimuli and *v*_max_ = 2°/s for 0.2 Hz. No feedback was provided as to the correctness of the responses after each trial. On only one test (1-Hz roll tilt) did the subject increase the stimulus amplitude beyond the motion device motion capabilities (1 out of more than 500 successful tests). When this occurred, since we thought that the subject may not have understood how to indicate the tilt direction, the subject was instructed again and given a second chance and then successfully completed the testing.

As a subtle enhancement to the published methods, all subjects used a two-stage task on an iPad to indicate responses. The iPad backlight illumination was off during all motion stimuli. Subjects were instructed to first tap the left (top) side of the screen if they perceived a leftward (upward) motion or to tap the right (bottom) side for rightward (downward) motion. Each tap was followed by feedback confirming the selection. Subjects were instructed that they must provide an answer. These instructions mimicked our earlier instructions, with the only difference as the use of an iPad instead of buttons to provide the binary indications. These standard binary data are used for all analyses presented herein.

After indicating perceived motion direction, subjects were instructed to indicate whether they were uncertain or not uncertain. If uncertain, subjects pressed the left and right sides of the iPad screen simultaneously. Otherwise, they pressed the same side of the screen again (e.g., right side twice for a right/certain response). These certainty/uncertainty data are not presented herein and are described here only to report our exact procedures.

As noted by others (13), testing at different frequencies could yield different results, especially since thresholds vary with frequency [e.g., Ref. (7, 8, 20, 26, 31–33)]. Roll tilts at 0.2 Hz were chosen to assess sensory integration between canal and otolith cues (34), but we chose 1-Hz stimuli for most testing because (1) subjects report that tasks using 1-Hz stimuli are easier than both (a) higher frequency (e.g., 5 Hz) stimuli that require high alertness to avoid missing brief stimuli and (b) lower frequency (e.g., 0.1 Hz) stimuli that require extended periods of attention and (2) they require just 1 s, so 100 trials can be accomplished in less than 10 min (including time for responses and pauses between trials).

### Data Analysis

For all conditions, the threshold (σ, sometimes called the psychometric width parameter) was determined by fitting a psychometric curve to the binary (e.g., left/right) experimental data. Specifically, a Gaussian cumulative distribution psychometric function defined by the parameters σ and μ was fit using a maximum likelihood estimate *via* a bias-reduced generalized linear model (BRGLM) (35) and probit link function (36). Fits were performed in MATLAB using the Statistic Toolbox version 8.3.

Geometric means were calculated for across subject averages, because, consistent with earlier reports (7, 8), data demonstrated a lognormal distribution across subjects for all conditions (Kolmogorov–Smirnov goodness-of-fit for lognormal distribution, *p* > 0.25). Both non-parametric and parametric analyses (using data in logarithmic units) were used. Multiple logistic regression was used to estimate the odds of failing the balance test associated with thresholds and age. A Pearson correlation was used to test for correlation between thresholds in different axes. Analyses were performed using SAS statistical software (SAS Institute Inc., Cary, NC, USA).

**Figure 1:**
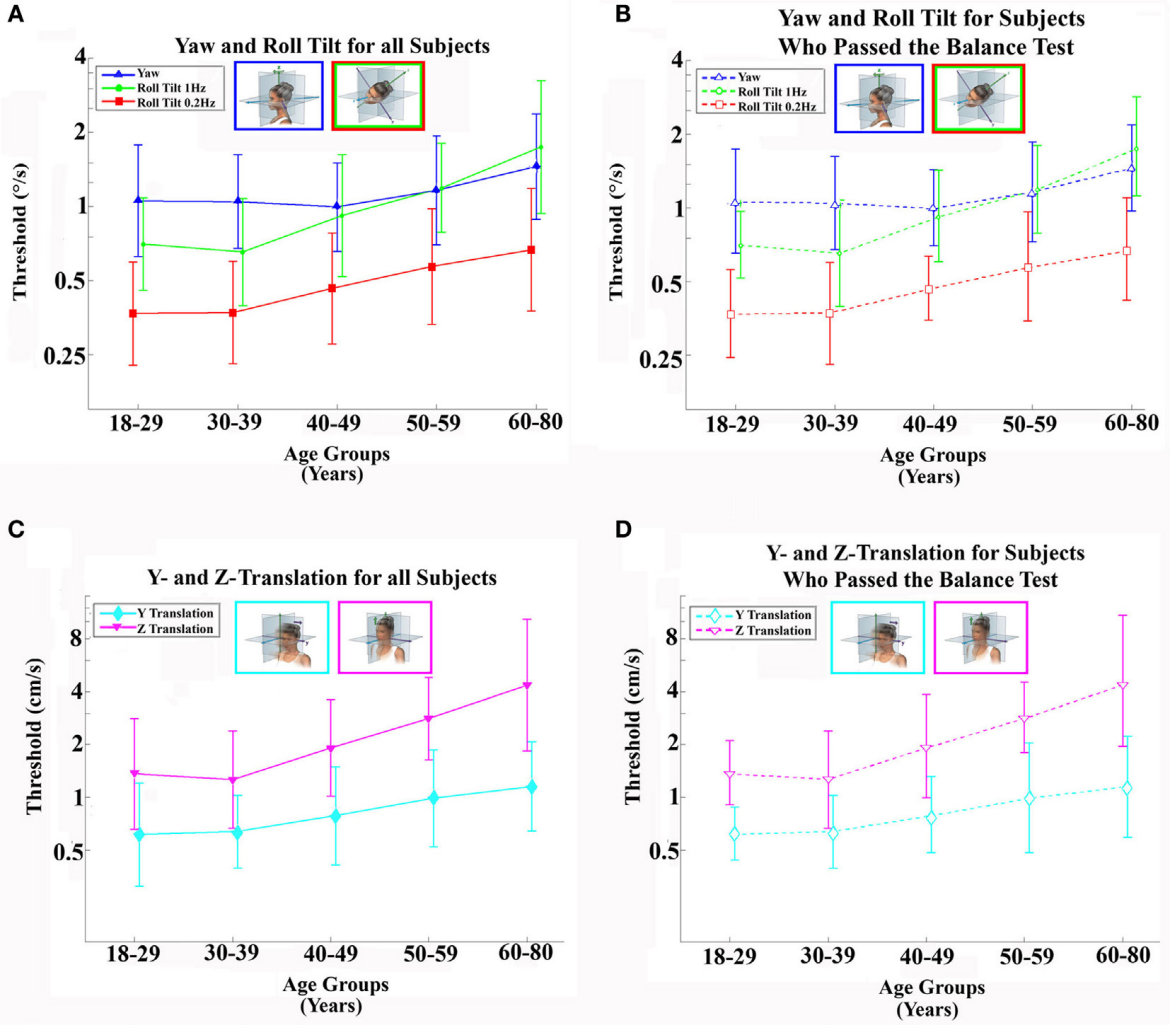
**Average (geometric mean) vestibular perceptual thresholds when grouped into five age ranges; error bars represent SD**. **(A,B)** Top row shows thresholds for 1-Hz yaw rotation (blue triangle), 1-Hz roll tilt (green circle), 0.2-Hz roll tilt (red square); **(C,D)** bottom row shows thresholds for 1-Hz *z*-translation (magenta triangle) and 1-Hz *y*-translation (cyan diamond). **(A,C)** Left column, with solid lines and filled symbols, represents data from all 105 subjects. **(B,D)** Right column, with dashed lines and open symbols, represents data from 79 subjects who completed and passed all steps of the balance test. For clarity, data points are offset left/right slightly to minimize overlap. Inset cartoons indicating motion direction are reprinted with permission from Wolfe et al. (40).

Data in other sensory domains [e.g., odor identification (37), visual acuity (38), and speech intelligibility (39)] suggest thresholds vary with age in a piecewise manner – with a flat plateau below an age cutoff and decreasing sensitivity above the same age cutoff. As our data shown in Figure 1 also suggest a similar piecewise linear pattern, we hypothesize thresholds remain relatively constant (i.e., no effect of age) up until some age cutoff at which point they increase (for simplicity, we assume this increase is linear). For each motion condition, the following continuous, piecewise linear model was fit to each subject’s threshold (σ*_i_*) data with three parameters: (1) an “age cutoff” $\left({\hat{a}}_{\text{cutoff}}\right)$, (2) a “baseline” level $\left({\hat{\sigma }}_{\text{baseline}}\right)$ that represents the average threshold for ages less than the age cutoff, and (3) a “slope” $\left(\hat{m}\right)$ that represents the rate of threshold increase above the age cutoff, where *a_i_* is each subject’s age in years rounded to the nearest integer at the time of testing was (e.g., 38 years of age). We present slope per decade (i.e., 10 years) throughout, since decades provide a more meaningful timescale for such changes.

$$\sigma_{i}=f(a_{i})=\left\{\begin{array}{cc} {\hat{\sigma }}_{\text{baseline}} & \text{if }a_{i}\leq {\hat{a}}_{\text{cutoff}}\\ \hat{m}\left(a_{i}-{\hat{a}}_{\text{cutoff}}\right)+{\hat{\sigma }}_{\text{baseline}} & \text{if }a_{i}>{\hat{a}}_{\text{cutoff}} \end{array}\right\}$$

As previously discussed, the thresholds were lognormally distributed; thus, the threshold data were log transformed and then a log-transformed version of the above age model was fit using a least-squared Nelder–Mead non-linear minimization routine (MATLAB fminsearch.m). Residuals were analyzed to assess the appropriateness of the fits. A parametric bootstrap approach (41), with *M* = 2,000 simulated data sets, was used to estimate the 95% confidence intervals of each fit parameter.

As shown in Figure 1, the age cutoffs were found to be similar across motion conditions. To quantify a single overall age cutoff, a comprehensive model (same piecewise form as above) was fit to the thresholds across all motion conditions. The model consisted of 11 parameters: 1 overall age cutoff, 5 baseline levels, and 5 slopes (corresponding to each of the 5 motion conditions). Each individual threshold data point was first log transformed, then standardized by the motion condition using the respective mean and SD prior to fitting a log-transformed version of the linear age model described above to each of the five data sets simultaneously. The standardization and log transformation processes were reversed to present model fit parameters and curves in the original physical units. We used likelihood ratio tests and Bayesian information criteria (BIC) to assess goodness-of-fit for the proposed piecewise two-segment linear models compared to alternative simple linear and average models.

## Results

### Thresholds

Our data do not suggest any threshold differences between males and females (Table 1). Statistical tests fail to demonstrate any significant effect of sex on thresholds. Even when we included migraine status, age, and balance test results as factors in multivariate analyses, no significant sex effect was found (*p* > 0.4, for each motion condition).

**Table 1:** **Thresholds for males and females (95% CI) for each of the five motion conditions**.

	Sex		Statistical analyses
	Male	Female	Wilcoxon rank sum
No. of participants	51	54
Yaw rotation (°/s)	1.05 (0.91–1.20)	1.18 (1.04–1.35)	*p* = 0.4474
*y*-translation (cm/s)	0.79 (0.66–0.96)	0.77 (0.65–0.91)	*p* = 0.8954
*z*-translation (cm/s)	1.84 (1.48–2.31)	2.09 (1.68–2.60)	*p* = 0.3992
Roll tilt 0.2 Hz (°/s)	0.47 (0.40–0.54)	0.45 (0.39–0.53)	*p* = 0.9463
Roll tilt 1 Hz (°/s)	0.91 (0.78–1.08)	0.94 (0.80–1.11)	*p* = 0.8450

*Data show no significant differences between sexes*.

We also looked for a potential difference between premenopausal women under hormonal contraception and normal cycling women (Table 2). In all conditions, women taking hormonal birth control had higher thresholds. This difference did not appear significant except for yaw rotation thresholds (Wilcoxon rank sum, *p* = 0.037). Given multiple comparisons, we do not treat this difference as significant.

**Table 2:** **Thresholds for females who are and are not taking hormonal birth control for each of the five motion conditions**.

	Hormonal birth control		Statistical analyses
	No	Yes	Wilcoxon rank sum
No. of participants	20	14
Yaw rotation (°/s)	0.92 (0.75–1.14)	1.32 (1.00–1.74)	*p* = 0.0373
*y*-translation (cm/s)	0.56 (0.47–0.68)	0.80 (0.52–1.24)	*p* = 0.1779
*z*-translation (cm/s)	1.32 (1.03–1.69)	1.65 (1.09–2.52)	*p* = 0.4732
Roll tilt 0.2 Hz (°/s)	0.35 (0.29–0.43)	0.37 (0.28–0.48)	*p* = 0.3303
Roll tilt 1 Hz (°/s)	0.67 (0.55–0.82)	0.80 (0.65–0.98)	*p* = 0.3359

*After multiple comparisons correction, no significant differences were found. 95% confidence intervals provided in parentheses*.

Furthermore, given that the association between hormonal contraception and yaw rotation thresholds could be explained by shared associations with other factors such as age, migraine status, or balance test results, we included all these factors in a multivariate analysis and found no significant effect of hormonal contraception on yaw rotation thresholds (*p* = 0.68) or for any of our other motion conditions.

In our sample, participants with migraine, defined as a MS-Q score of 4 or more (23), had lower thresholds for 4 of the 5 conditions – all but yaw rotation – than all other subjects. After correcting for multiple comparisons, that potential difference was not significant (Table 3). Because the sample size for migraine sufferers was so low (*N* = 5), this is noted as interesting but was not further explored herein.

**Table 3:** **Threshold dependent on migraine status (95% CI) for each of the five motion conditions**.

	Migraine status		Statistical analyses
	No	Yes	Wilcoxon rank sum
No. of participants	100	5
Yaw rotation (°/s)	1.11 (1.01–1.22)	1.16 (0.66–2.01)	*p* = 0.9221
*y*-translation (cm/s)	0.80 (0.71–0.91)	0.46 (0.34–0.62)	*p* = 0.0462
*z*-translation (cm/s)	2.03 (1.73–2.38)	1.05 (0.87–1–27)	*p* = 0.0430
Roll tilt 0.2 Hz (°/s)	0.47 (0.42–0.52)	0.31 (0.25–0.39)	*p* = 0.0698
Roll tilt 1 Hz (°/s)	0.94 (0.83–1.06)	0.76 (0.48–1.21)	*p* = 0.4122

*Significant differences were suggested for z-translation and y-translation, but after multiple comparisons correction, no significant differences were found*.

Table 4 shows the velocity threshold geometric mean for each motion condition separated into five age groups. As previously reported (7), *z*-translation thresholds were significantly higher (typically ~2× higher) than *y*-translation thresholds (paired *t* test, *p* < 0.0001). Yaw rotation thresholds were higher than roll tilt 1-Hz thresholds (paired *t* test, *p* = 0.0028), and roll tilt 0.2-Hz thresholds were significantly lower than both yaw rotation and roll tilt 1-Hz thresholds (paired *t* test, *p* < 0.0001 each). All five motion conditions showed an increase of threshold (poorer direction-recognition performance) with age (Figure 1). We note that all five subplots show a relatively flat threshold plateau below the age of 40–49 and also show increasing thresholds above that same age cutoff.

**Table 4:** **Mean threshold by age group for each of the motion conditions, with a 95% confidence interval**.

Age (in years)	No. of subjects	Yaw rotation (°/s)	*y*-translation (cm/s)	*z*-translation (cm/s)	Roll tilt 0.2 Hz (°/s)	Roll tilt 1 Hz (°/s)
All	105	1.11 (1.01–1.23)	0.78 (0.69–0.89)	1.97 (1.68–2.30)	0.46 (0.41–0.51)	0.93 (0.83–1.04)
18–29	29	1.06 (0.87–1.28)	0.61 (0.48–0.79)	1.36 (1.04–1.77)	0.37 (0.31–0.44)	0.70 (0.60–0.82)
30–39	20	1.04 (0.86–1.26)	0.64 (0.52–0.78)	1.26 (0.96–1.67)	0.37 (0.30–0.46)	0.65 (0.52–0.81)
40–49	19	0.99 (0.83–1.19)	0.79 (0.59–1.05)	1.91 (1.44–2.53)	0.46 (0.37–0.59)	0.92 (0.71–1.18)
50–59	21	1.16 (0.94–1.44)	0.99 (0.75–1.29)	2.81 (2.23–3.53)	0.57 (0.45–0.72)	1.19 (1.00–1.42)
60–80	16	1.45 (1.14–1.84)	1.15 (0.87–1.53)	4.35 (2.86–6.60)	0.67 (0.51–0.88)	1.74 (1.29–2.35)
Passed balance	79	1.04 (0.94–1.16)	0.69 (0.61–0.79)	1.62 (1.38–1.91)	0.40 (0.36–0.45)	0.81 (0.72–0.91)
18–29	24	0.98 (0.79–1.21)	0.51 (0.43–0.60)	1.14 (0.93–1.40)	0.34 (0.29–0.41)	0.63 (0.55–0.73)
30–39	20	1.04 (0.86–1.26)	0.64 (0.52–0.79)	1.26 (0.95–1.67)	0.37 (0.30–0.46)	0.65 (0.52–0.81)
40–49	13	0.87 (0.70–1.09)	0.70 (0.52–0.95)	1.74 (1.17–2.62)	0.39 (0.32–0.47)	0.81 (0.62–1.06)
50–59	14	1.16 (0.91–1.48)	0.97 (0.66–1.42)	2.43 (1.83–3.21)	0.52 (0.38–0.70)	1.17 (0.94–1.46)
60–80	8	1.37 (1.02–1.85)	1.18 (0.75–1.85)	3.80 (1.91–7.59)	0.58 (0.39–0.85)	1.45 (0.98–2.14)

**Figure 2:**
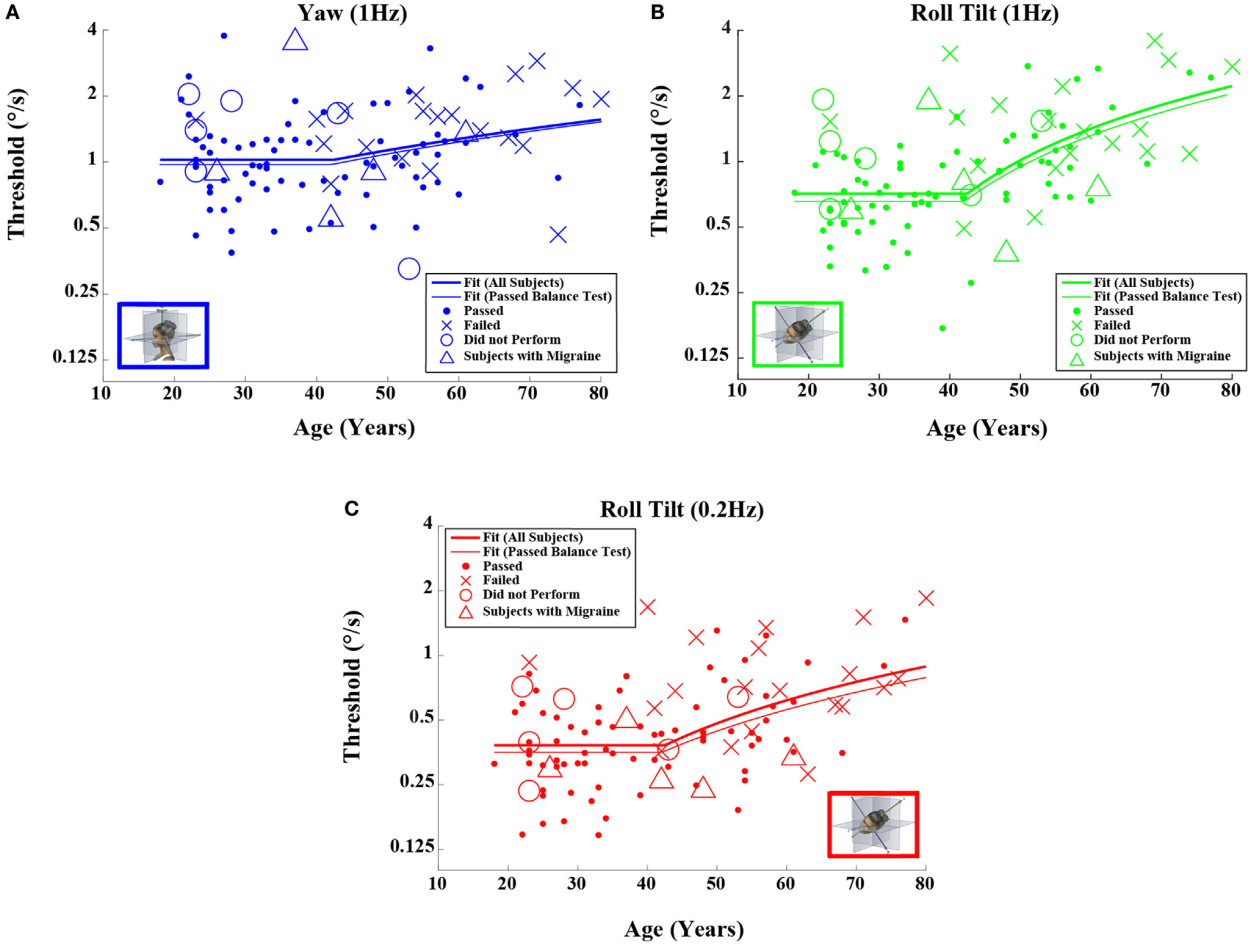
**Threshold data for all subjects are plotted versus age for (A) 1-Hz yaw rotation, (B) 1-Hz roll tilt, and (C) 0.2-Hz roll tilt**. Closed circles (●) show data for subjects who passed the balance test. Cross mark (X) show data for 20 subjects who passed conditions 1–3 but did not pass condition 4 of the balance test. Open circles (○) show data for six subjects who did not attempt the balance test. Triangles (Δ) show data for five migraineurs. Inset cartoons indicating motion direction are reprinted with permission from Wolfe et al. (40).

**Figure 3:**
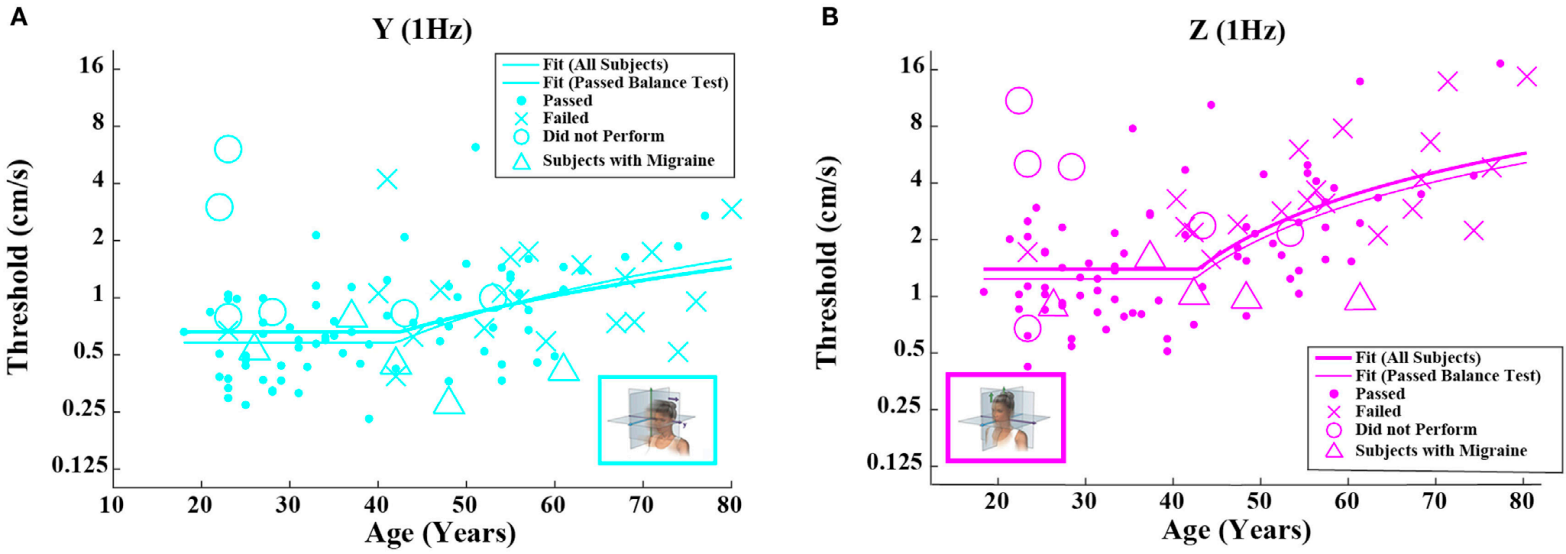
**Threshold data for all subjects are plotted versus age for (A) 1-Hz *y*-translation and (B) 1-Hz *z*-translation**. Closed circles (●) show data for subjects who passed the balance test. Cross mark (X) show data for 20 subjects who passed condition 3 but did not pass condition 4 of the balance test. Open circles (○) show data for six subjects who did not attempt the balance test. Triangles (Δ) show data for five migraineurs. Inset cartoons indicating motion direction are reprinted with permission from Wolfe et al. (40).

When each of the five motion conditions was analyzed separately, this age effect was significant, even following multiple comparisons correction, for four of the five motions tested (Kruskal–Wallis, *p* < 0.005) – all but yaw rotation. A similar trend with age was evident in the yaw rotation data, but this trend was not statistically significant (Kruskal–Wallis, *p* = 0.087). Figures 2 and 3 show these data.

Having established that there is an age effect that is independent of other factors, we evaluated whether there is an age cutoff above which threshold increases accumulate by fitting a two-piece linear model to the data. To minimize the impact of undiagnosed vestibular dysfunction, this model fit was first performed only on data from subjects who passed the balance test. Fitting this model to the data set for each motion individually (Table 5), we found an average age cutoff of 42.6 years. The residuals were consistent with a normal distribution (KS tests, *p* > 0.4).

**Table 5:** **Fit parameters determined by fitting each motion condition individually for subjects who passed the balance test**.

Motion	Age cutoff (years)	Baseline	Slope (per decade)	Slope (% per decade)
Yaw rotation 1 Hz	46.2 (24.3–65.8)	0.98 (0.82–1.10) °/s	0.19 (0.04–0.94) °/s	19.56 (15.35–23.77)
*Y*-translation 1 Hz	39.0 (26.6–50.0)	0.57 (0.47–0.67) cm/s	0.23 (0.12–0.54) cm/s	40.33 (20.29–60.37)
*Z*-translation 1 Hz	42.0 (32.2–49.3)	1.24 (1.02–1.46) cm/s	1.02 (0.52–2.20) cm/s	82.80 (42.12–123.47)
Roll tilt 1 Hz	43.0 (32.2–49.7)	0.66 (0.57–0.74) °/s	0.39 (0.20–0.72) °/s	59.77 (28.42–91.11)
Roll tilt 0.2 Hz	42.6 (27.0–54.0)	0.35 (0.30–0.40) °/s	0.12 (0.05–0.33) °/s	33.32 (18.46–48.18)

*95% confidence intervals provided for each parameter in parenthesis*.

Given that the fitted “age cutoff” was similar across the motion conditions, we also fit a model having 11 parameters that fit a single age cutoff across all 5 threshold data sets while simultaneously fitting 2 parameters (slope above cutoff age, baseline below cutoff age) to each of the 5 motion conditions. This fit was performed twice – once with all of the data and once with data obtained from subjects who passed the balance test. Table 6 shows the results from this fit. As can be seen in Figures 2 and 3 and Table 6, the two fits yielded similar curves. The overall age cutoff when fit simultaneously across all motion conditions was 42.1 years for all subjects and 42.7 years for all subjects who passed the balance test. Each of the slope values shown in Table 6 was significantly greater than 0 (*p* < 0.05) corresponding to an increase in threshold (worse performance) with increasing age above ~40 years.

**Table 6:** **Fit parameters determined *via* single simultaneous fit of all threshold data**.

Motion	Baseline	Slope (per decade)	Slope (% per decade)
**All subjects**			
Yaw rotation 1 Hz	1.02 (0.93–1.13) °/s	0.14 (0.04–0.27) °/s	14.0 (3.9–27.5)
*Y*-translation 1 Hz	0.66 (0.57–0.76) cm/s	0.21 (0.10–0.35) cm/s	31.6 (14.2–57.0)
*Z*-translation 1 Hz	1.39 (1.15–1.66) cm/s	1.17 (0.68–1.87) cm/s	84.1 (45.0–141.9)
Roll tilt 1 Hz	0.71 (0.61–0.81) °/s	0.40 (0.24–0.61) °/s	56.6 (32.9–90.1)
Roll tilt 0.2 Hz	0.38 (0.34–0.43) °/s	0.14 (0.07–0.21) °/s	35.4 (18.6–58.7)
**Passed balance test**			
Yaw rotation 1 Hz	0.97 (0.87–1.09) °/s	0.15 (0.02–0.31) °/s	14.9 (01.9–34.5)
*Y*-translation 1 Hz	0.58 (0.50–0.67) cm/s	0.27 (0.13–0.47) cm/s	46.0 (20.5–85.6)
*Z*-translation 1 Hz	1.24 (1.02–1.49) cm/s	1.03 (0.56–1.86) cm/s	83.2 (42.3–160.0)
Roll tilt 1 Hz	0.66 (0.57–0.74) °/s	0.37 (0.21–0.61) °/s	56.0 (30.5–95.3)
Roll tilt 0.2 Hz	0.35 (0.31–0.40) °/s	0.11 (0.05–0.20) °/s	32.4 (13.9–58.4)

*95% confidence intervals provided for each parameter in parenthesis. Top half of the table shows fitted parameters when all data are fitted; fitted age cutoff was 42.5 (37.1–46.9). Bottom half shows fitted parameters for subjects who passed the balance test; fitted age cutoff was 42.1 (36.5–46.6)*.

Table 7 shows fitted degrees of freedom (DOF), variance explained, −2 × log(likelihood), and BIC for each of the four different models presented: mean model, simple linear model, simultaneous two-segment linear model, and the independent two-segment linear model. To allow us to provide a single estimate of variance and BIC for each fitting method, we emphasize that each of the five data sets were standardized using customary calculations (i.e., the mean was subtracted from each data point and then divided by SD) described earlier in the methods, which yields two benefits. First, it makes all parameters dimensionless, which allows us to combine variance across different motion conditions. Second, it makes the variance for each of our five motion dimensions the same, which provides even weighting across the five measures (Otherwise, the variance could be dominated by the measure having the greatest variance).

**Table 7:** **Degrees of freedom (DOF), variance explained, −2 × log(likelihood) – which is sometimes called deviance and was calculated using the natural log – and Bayesian information criteria (BIC) scores are shown for the four different models presented herein**.

Name	DOF	% variance explained	−2 log(*L*)	BIC
**All subjects (*N* = 105)**				
Mean	5	–	−4.02	27.29
Linear regression	10	12.10	−71.8	−9.21
Simultaneous 2-segment	11	20.70	−125.8	−56.95
Independent 2-segment	15	20.90	−126.7	−32.85
**Passed balance test (*N* = 79)**				
Mean	5	–	−4.03	25.86
Linear regression	10	7.20	−33.6	26.15
Simultaneous 2-segment	11	20.10	−92.6	−26.79
Independent 2-segment	15	20.30	−93.6	−3.92

*The simplest model simply calculates the mean (e.g., Table 1), ignoring age variations, and provides a standard statistical baseline for the three other models. The linear regression model (Table 5) has 10 fit parameters – a slope and intercept for each of the 5 motion directions. The independent 2-segment model has 15 fit parameters – a baseline, age cutoff, and slope for each of our 5 threshold measures reported herein. The simultaneous 2-segment model has 11 fit parameters – a single age cutoff as well as a baseline measure and slope for each of 5 threshold measures. The BIC was the smallest (best) for the 11-parameter model both for all subjects and for subjects who passed the balance test*.

As one would expect, more fit parameters (i.e., more DOF) yields variance reductions, but the 11-parameter 2-segment model explains roughly twice the variance of the 10-parameter model. In other words, adding a single age cutoff parameter to the linear regression model doubles the variance explained.

Likelihood ratio testing was used to test the nested models (mean, simultaneous two-segment, and independent two-segment) and showed that the proposed simultaneous two-segment linear model was significantly better than the mean model for both the full data set (χ^2^ statistic = 121.8, DOF = 6, *p* < 0.0001) and the 79 subjects who passed the balance test (χ^2^ statistic = 88.6, DOF = 6, *p* < 0.0001). Likelihood ratio testing also showed the simultaneous two-segment linear model is not significantly different from the independent two-segment linear model for both the full data set (χ^2^ statistic = 0.9, DOF = 4, *p* = 0.92) and the 79 subjects who passed the balance test (χ^2^ statistic = 1.0, DOF = 4, *p* = 0.91).

Bayesian information criteria statistics showed that this model was substantially better than the other models considered, including the simple linear model. In fact, this analysis showed that the simultaneous two-segment model had the smallest BIC for both the full data set and for the 79 subjects who passed the balance test. The BIC for the 11-parameter 2-segment model was always more than 20 points lower than for any other model. For context, BIC differences greater than 10 are considered “very strong” evidence for the model with the lower BIC (42).

The two simple models – the mean and linear regression models – do not match our data well. The mean model cannot capture the fact that thresholds increase above the age of about 40, and the linear regression model cannot capture the fact that thresholds are relatively constant below the age of about 40 (e.g., Figure 1).

### Bias

For our direction-recognition task, the fitted bias parameter represents the stimulus magnitude at which a subject is equally likely to respond right (up) or left (down) (43). It is poorly understood because it could originate from any of the three sources: (a) a bias in the information, (b) a bias in the placement of the decision boundary (43), or (c) a bias in the noise distribution. We evaluated bias and normalized bias; normalized bias is simply the fitted bias divided by the fitted threshold. This dimensionless parameter has the advantage that it is readily comparable across motion conditions.

Neither the mean value of the bias nor the mean value of normalized bias was significantly different from 0 for any of the 5 motion conditions. This was true across all 105 subjects as well as for the 79 subjects who passed the balance test (*t* test, *p* > 0.05 for all 20 conditions tested after correction for multiple comparisons).^1^ Furthermore, correlation coefficients for either bias or normalized bias versus age were not significantly different from 0 for any of the 5 motion conditions. Again, this was true for all subjects as well as for those who passed the balance test (Kruskal–Wallis, *p* > 0.1 for all 20 conditions tested).

**Figure 4:**
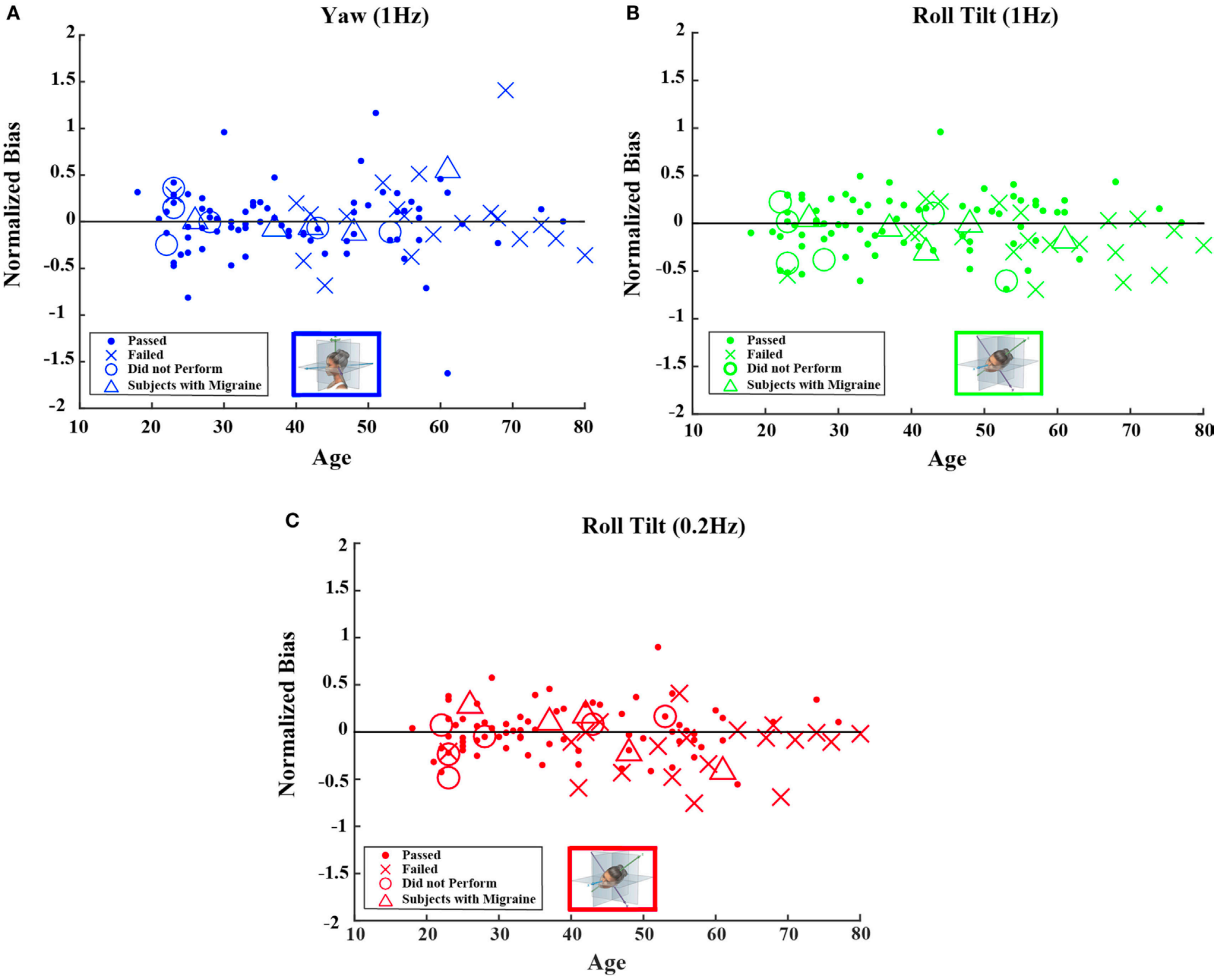
**Normalized bias data for all subjects are plotted versus age for (A) 1-Hz yaw rotation, (B) 1-Hz roll tilt, and (C) 0.2-Hz roll tilt**. Closed circles (●) show data for subjects who passed the balance test. Cross mark (X) show data for 20 subjects who passed condition 3 but did not pass condition 4 of the balance test. Open circles (○) show data for six subjects who did not attempt the balance test. Triangles (Δ) show data for five migraineurs. Format mimics Figure 2.

**Figure 5:**
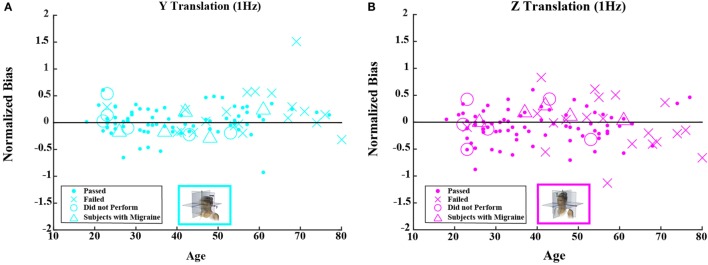
**Normalized bias data for all subjects are plotted versus age for (A) 1-Hz *y*-translation and (B) 1-Hz *z*-translation**. Closed circles (●) show data for subjects who passed the balance test. Cross mark (X) show data for 20 subjects who passed condition 3 but did not pass condition 4 of the balance test. Open circles (○) show data for six subjects who did not attempt the balance test. Triangles (Δ) show data for five migraineurs. Format mimics Figure 3.

Figures 4 and 5 show scatterplots of the normalized bias values for all the motion conditions. Consistent with correlation analyses reported above, no significant or consistent trends are evident.

### Relationship between Thresholds and Romberg Balance Testing

Given that vestibular information is a fundamental contributor to balance control, we looked for associations between thresholds and performance on the modified Romberg test. A subsample of 99 subjects performed the balance test. Not a single participant failed the test in conditions 1, 2, or 3, but 20% (20 of 99) failed in condition 4. Thresholds were significantly greater for participants who failed the final balance test condition (i.e., the Romberg test condition focused primarily on vestibular function) for all motion axes (Table 8).

**Table 8:** **Mean thresholds for subjects who passed and failed the balance test**.

	Balance test		Statistical analyses
	Pass	Fail	Wilcoxon rank sum
No. of participants	79	20	
Yaw rotation (°/s)	1.04 (0.94–1.16)	1.43 (1.19–1.71)	*p* = 0.0030
*y*-translation (cm/s)	0.69 (0.61–0.79)	1.05 (0.81–1.35)	*p* = 0.0066
*z*-translation (cm/s)	1.62 (1.37–1.91)	3.67 (2.79–4.84)	*p* < 0.0001
Roll tilt 0.2 Hz (°/s)	0.40 (0.36–0.45)	0.76 (0.60–0.95)	*p* < 0.0001
Roll tilt 1 Hz (°/s)	0.81 (0.72–0.91)	1.55 (1.18–2.03)	*p* < 0.0001

*Significant differences were found for each of the motion conditions*.

Consistent with earlier findings (25), the proportion of balance test failures increased with age group (Fisher exact test, *p* < 0.0001). To test whether the observed difference between the pass and fail group may be due to an age effect or to the influence of other confounding factors, we adjusted for age, gender, and migraine using a mixed model. The significant association of balance test failure with increasing threshold remained persistent for roll tilt at both 0.2 and 1 Hz (*p* = 0.003 and *p* = 0.02, respectively) but was not significantly correlated for increasing yaw rotation (*p* = 0.09), *y*-translation (*p* = 0.50), and *z*-translation (*p* = 0.09) thresholds. The age effect was unchanged for all motion paradigms – remaining significant for *y*-translation, *z*-translation, and roll tilt at 1 Hz (each *p* < 0.0001) and roll tilt at 0.2 Hz (*p* = 0.0007) and not significant for yaw rotation (*p* = 0.12).

Odds of failing the balance test have been associated with significantly increased odds of falling, for American adults 40 years and older (25). Our data show that a 1 unit increase in roll-tilt thresholds at 0.2 Hz (following transformation in SAS using natural log) were associated with a 5.6-fold increase in the odds of failing the balance test (odds ratio, 5.6; 95% confidence interval, 1.6–18.9) in a multiple logistic regression adjusted for age. One-unit increase in roll-tilt thresholds at 1 Hz (log-transformed version) was associated with a 3.7-fold increase in the odds of balance testing failure (odds ratio, 3.7; 95% confidence interval, 1.1–12.9).

## Discussion

In this study, we attempted to determine whether sex or age affected perceptual thresholds of vestibular functioning. To do this, we asked subjects to indicate the direction of movement they perceived in 5 blocks of 100 trials each for the following motion conditions: yaw rotation, *y*-translation, *z*-translation, roll tilt at 1 Hz, and roll tilt also at 0.2 Hz. One primary finding was that thresholds increased with age for all motion directions above the age of about 40. This finding is consistent with a number of earlier threshold studies that had reported similar effects of age *x*-axis translation (16, 19) and *y*-axis translation (16) but inconsistent with a few earlier studies that reported no such age effects for yaw rotation (14–16). We note that the yaw rotation age effect was the smallest that we observed and our study had a larger total number of subjects than the earlier studies; these differences likely explain why we found a significant effect of aging in contrast to earlier studies.

We explicitly note that our finding of a statistically significant effect of age on yaw rotation thresholds required both our two-segment model and more than 50 subjects. Our translation threshold findings showed more substantial age effects. These findings are consistent with earlier findings that translation thresholds increase with age (16–19). No previous studies have examined roll-tilt thresholds, which is a focus of our study (i.e., 40% of the data reported).

The second primary finding was that increasing roll-tilt threshold was correlated with failure to complete the Romberg foam balance test. Since we know of no mechanism by which balance would impact vestibular thresholds, and since we know that balance depends on vestibular function [e.g., Ref. (44)] and that falls correlate with failure to complete the Romberg foam balance test (25), it is reasonable to suggest that this correlation shows that fall risk is substantially impacted by vestibular function. We emphasize that this was true even when measured in a healthy population chosen without any evidence of specific vestibular disorders.

Finally, while we did not report a full analysis of the within subject correlations between thresholds in different axes, our initial analyses showed that 9 of the 10 pairwise comparisons (all but the correlation of yaw rotation thresholds with *y*-translation thresholds) of the 5 threshold measurements were correlated (*p* < 0.05); we plan to make this topic the focus of a future manuscript as soon as we complete extensive analyses of these correlations. A more detailed discussion of our findings and the implications are presented below.

### Sex Differences and Individual Differences

One goal when conducting this study was to determine whether there were sex differences in vestibular perceptual thresholds. According to our findings, there were no differences between males and females in their perceptual thresholds; this finding is in line with other studies comparing sex differences (8, 16, 17, 26).

Benson and colleagues previously published a pair of comprehensive threshold studies (7, 8). These studies looked at rotational thresholds (8) and translational thresholds (7). For the 4 motions studied with 24 or more subjects, all demonstrated a range of thresholds with a ratio of roughly 10 for all 4 motions (i.e., the maximum threshold divided by the minimum threshold was about 10). In decibel units, Benson reported SDs of 4.40 (yaw rotation), 4.63 (*X*-translation), 3.98 (*Y*-translation), and 6.10 (*Z*-translation). When converted to decibels our experimental SDs were 4.11 (yaw rotation), 5.23 (*Y*-translation), 6.47 (*Z*-translation), 4.62 (1.0-Hz roll tilt), and 4.36 (0.2-Hz roll tilt), which appear similar to Benson’s. These similar empiric variations reported by both studies are most likely due to intersubject differences since variations due to sampling and other methodological details have been shown to be an order of magnitude smaller for 100 binary forced-choice trials (29) than these empirical variations.

### Motion Differences

Next, we looked at differences in perceptual thresholds for the various motion stimuli. We found differences in several of the motion thresholds that we tested. Namely, we found that the threshold for *y*-translation was less than the threshold for *z*-translation, by approximately a factor of 2. This finding is consistent with MacNeilage et al. who indicated that utricles may have a greater sensitivity to perceive horizontal motion compared to the saccules’ sensitivity to perceive vertical motion (45). This suggestion carries more weight when we also consider that the utricles have a greater density of hair cells compared to the saccules (46). It is possible that the additional hair cells in the utricles could contribute to their sensitivity and therefore lower the *y*-threshold. Furthermore, Valko et al. suggest that earth-vertical movement is more difficult to discriminate as the otolith must determine whether the gravitational force increases or decreases, whereas for an earth-horizontal movement, the body and brain have access to a greater amount of non-vestibular cues to aid in determining the direction of a particular motion (20). Overall, there are many differences between earth-vertical and earth-horizontal movements; it is possible that the amount of information (e.g., tactile) available during earth-horizontal movement provides easier perception and recognition of the motion direction compared to the amount of information available when moving along the *z*-axis.

When we divided the data by age group, the threshold velocity for roll tilt at 0.2 Hz was less than the threshold velocity for 1-Hz roll tilt. This is consistent with Valko et al.’s study, where the researchers found that roll-tilt thresholds, expressed as peak velocity, decreased at lower frequencies in healthy subjects (20).

Furthermore, the threshold for roll tilt at 1 Hz was also lower than the yaw threshold. Because roll tilt is perceived by an integration of otolith and semicircular canals, the amount of information available to the vestibular system may facilitate the perception of motion compared to the yaw rotation which primarily relies on the semicircular canals.

### Ages for Perceptual Cutoffs

Finally, because we saw that thresholds for each motion condition increased from decade to decade after about age 40, we thought it would be interesting to determine whether there was one specific age at which the perceptual thresholds began to increase for all five motion paradigms. By modeling the data we collected in this study, we found that we were able to separate vestibular thresholds into two categories, namely, “younger” and “older” adults, where younger adults’ thresholds were stable until about 40 years of age, at which point “older” adult vestibular performance began to decline (i.e., thresholds began to increase) at a steady rate for each of the motion thresholds. Between this finding and findings from other studies (16, 18, 47), we can directly assert that changes in vestibular function occur with age. While increasing age above the age cutoff was associated with an increase in threshold in each motion condition, some conditions were impacted more than others: *z*-translation thresholds increased by ~83% of the baseline per decade after the age cutoff, roll tilt 1 Hz by 56%/decade, *y*-translation by 46%/decade, roll tilt 0.2 Hz 32%/decade, and yaw rotation 15%/decade. These rates of increased thresholds with aging are for the subset of subjects that passed the balance test, but the values when including all subjects are similar.

### Vestibular System Aging

Vestibular functioning can decline for any number of reasons including neurodegenerative disease, peripheral loss, and even medications and their side effects. Age is an important factor that influences the vestibular system and vestibular function. While the mechanisms behind aging remain disputed, it is an important and relevant issue to address here. While others have considered the mechanisms of aging and its particular effect on the vestibular system (48), we would like to expand on what has previously been proposed by comparing age-related changes in the vestibular system to other systems, by discussing potential mechanisms, and consider why little or no threshold difference occur before the age of 40.

#### Comparison to Other Modalities

We report that – for five different tests of vestibular function – perceptual thresholds appeared constant between the ages of 20 and 40 and increased linearly above the age of 40. A roughly similar pattern has been reported for other sensory systems but with the functional performance plateau lasting until the age of 60. For example, average odor identification shows a plateau until about the age of 60 with functional declines evident above the age of 60 (37). As shown in Figure 2 of Doty, visual acuity (38) and speech intelligibility (39) show similar patterns including declines above the age of about 60. It is interesting to note that the functional decline appears to begin about two decades earlier for vestibular function than for smell, vision, or speech intelligibility. This may indicate that vestibular function is preferentially targeted by whatever mechanism(s) causes functional sensory loss with age (e.g., vestibular threshold increases with age).

#### Mechanism

The threshold variations were qualitatively similar across all conditions; this suggests at least one shared common cause. The fact that the functional decline pattern (i.e., roughly linear threshold increase begins to occur around age 40 for all conditions) is about the same for all conditions tested weighs against an “overstimulation” cause like that reported for hearing loss (49–51), though that certainly does not mean that hearing loss and functional vestibular loss cannot share another (or other) mechanism(s).

No explanation, including our common cause explanation, can explain the quantitative differences across motions (e.g., why do *z*-translation thresholds demonstrate a slope of more 80% while yaw rotation a 15% slope?) at this time. One simple explanation is that these do not share a common cause. Alternatively, these deficits could reflect a common cause with the quantitative differences due to (a) the amount of available redundancy, which may vary for different motions, (b) the baseline, which obviously impacts this relative slope measure, and/or (c) other mechanisms in addition to a cause common to all motions.

Several studies have reported human vestibular hair cell and vestibular afferent neuron counts as a function of age (46, 52). Vestibular hair cell loss has been reported to show a linear decline with age from birth through 100 years of age that does not directly match our threshold data, especially the constant threshold plateau we report below age 40. This argues against hair cell loss in isolation being a direct explanation of the threshold age pattern we report. Similarly, the loss of afferent neurons does not in isolation match the threshold age pattern we report.

Therefore, we will briefly consider another (possibly related) cause – the free radical theory of aging, which is probably the most persistent theory of aging and could explain why performance declines begin to be evident for vestibular thresholds around the age of 40 but later for some other sensory functions. Specifically, we note that in primates, the average firing rate of peripheral afferent neurons is nearly 100 spikes per second, with the resting rate reported as averaging 91.3 spikes per second for the semicircular canals (53) and 62.7 spikes per second for the otolith organs – 79.1 for superior nerve and 47.0 for inferior nerve (54). These resting rates average between 50 and 100 spikes per second across about 40,000 vestibular afferent neurons (40) and, hence, assert a substantial metabolic load. While the resting rate for individual neurons in the vestibular nuclei is a bit lower, the central vestibular system similarly asserts a substantial metabolic load [e.g., Ref. (55)].

A metabolic-related cascade that could lead to age-related vestibular threshold increases is sketched in the following paragraphs. The heavy metabolic load of the vestibular system – both central and peripheral vestibular systems – requires extensive ATP production *via* mitochondria. Since mitochondria are the biggest contributors of oxidative load (i.e., free radicals) to the body, this leads directly to a relatively large oxidative stress. Many of these free radicals are quenched. Others escape and cause damage distributed elsewhere. But some of these free radicals cause local damage. This local damage can lead to dysfunctional central and/or peripheral function. This proposed mechanism would be consistent with studies showing oxidative contributions to peripheral cochlear dysfunction [e.g., Ref. (56)]. When the vestibular functional loss (i.e., neuronal cell death and/or dysfunction) leads to more “signal” loss than “noise” loss, it would lead to increased perceptual thresholds.

A review of the evidence for/against the free radical theory of aging and other theories of aging is beyond the scope of this paper but can be found in various books/reviews [e.g., Ref. (57–59)] We simply note here that the most recent incarnation (60, 61) of the free radical theory of aging (59, 62, 63) would be consistent with the cascade described above. If the free radical theory of aging is the major contributor to the deficit observed in thresholds above the age of 40, this could make vestibular threshold changes a relatively simple, sensitive, non-invasive behavioral biomarker for aging in humans above the age of 40, especially, since, as noted earlier, the age effects for vestibular function appear earlier than for odor discrimination, visual acuity, or speech intelligibility.

#### Why Are No Threshold Changes Evident below the Age 40?

Small threshold changes below the age of 40 may be evident for an individual but were masked by intersubject variability, since our study was not longitudinal. Long-term longitudinal studies would likely show whether threshold changes occur in individual humans before the age of 40.

While speculative, the free radical mechanism described earlier could also be consistent with the relative threshold constancy before age 40. Let us assume that vestibular contributions are crucial and that oxidative neuronal cell loss due to cell death or neuronal dysfunction is inevitable. If true, one reasonable evolutionary strategy would be to have an excess of neurons at least till reproductive vigor started to wane. In other words, some neuronal cell loss occurs before age 40, but the available excess of vestibular neurons yields redundancy such that the incremental decrease in overall signal matches the incremental decrease in overall noise for each vestibular neuron lost due to dysfunction or death. But around age 40, the vestibular cell counts reduce to the point that each neuron loss causes more incremental signal loss than noise loss. If true, this would suggest that we have on the order of 40,000 vestibular afferent neurons to provide some redundancy to fend off functional impact of peripheral vestibular loss. Such peripheral redundancy could also explain why thresholds as a function of age do not match the aging patterns shown by vestibular afferent neuron counts (52, 64) or vestibular hair cell counts as a function of age (46, 64). Furthermore, this hypothesis could account for different quantitative aging patterns reported herein (i.e., *Z*-translation slope much greater than yaw rotation slope relative to baseline) for different movements.

### Implications

Our threshold data showed that vestibular thresholds broadly increased with age above the age of 40 (by 15–83% per decade depending upon the motion condition, *p* < 0.05 for all five threshold measures). Furthermore, our data showed that balance test failures increased significantly as roll-tilt thresholds increased – even when age and other factors were fully considered by our mixed-model analysis. This latter finding is important because an earlier study showed that failure to complete the Romberg foam balance test correlates highly with falls (25). More specifically, data from a National Health and Nutrition Examination Survey (NHANES) were analyzed to show that 35.4% of American subjects above the age of 40 were unable to stand on foam with their eyes closed – the exact same failure that we showed to be significantly correlated with increasing roll-tilt thresholds. The earlier study (25) also reported increased odds of falling – an odds ratio of 6.3 – for such individuals with subclinical vestibular dysfunction relative to those without dysfunction (i.e., individuals who were able to complete the balance testing successfully).

Given different definitions and different methods, these American findings are neither far from the findings of a German study that estimated prevalence of vertigo to be 22.9% (65) nor from self-reported vertigo prevalence rates of about 20% (66, 67). In fact, self-reported dizziness for the American study was 27.0% – certainly in line with the earlier estimates. For the fraction of such symptomatic individuals with measured vestibular dysfunction, the American study reported increased odds of falling – i.e., an odds ratio of 12.3. Our findings of a decrement in vestibular function – directly assessed *via* vestibular thresholds – above the age of 40 could certainly help explain why 35% of the NHANES population above the age of 40 demonstrated balance dysfunction.

Given the clear evidence presented herein that vestibular function declines with age above the age of 40 and given the relative consistency of the earlier estimates of vestibular and balance dysfunction (25, 65–67), it seems reasonable to try to make a conservative estimate of the number of people who might die each year due to vestibular dysfunction. For example, it seems likely that at least some of the transportation accidents (e.g., car crashes) that lead to the death of about 50,000 Americans each year (39) are due to vestibular dysfunction, but, unfortunately, we were unable to find enough relevant data at this time to estimate the contributions of vestibular dysfunction to motor vehicle accidents. On the other hand, available data do allow us to conservatively estimate the number of deaths each year caused by falls related to vestibular dysfunction. These calculations are provided in detail in Appendix A. Table A1 in Appendix provides a range of estimates – some more conservative and some less so. The annual death estimates correlated with vestibular dysfunction range from 48,000 to 152,000.^2^ While the largest estimate of nearly 152,000 deaths per year may prove inaccurate, it is worth noting that this would be placed third in the US behind only heart disease and cancer. Even the lowest estimate of ~48,000 deaths per year – which would place this as the tenth largest cause of death in the US – conveys the gravity of the problem.

We emphasize that estimating death rates was not a goal of our study but rather an implication of the finding – even after correcting for age effects – that the proportion of balance test failures increased with roll-tilt thresholds, especially at 0.2 Hz (*p* = 0.0007); 1 unit increase in roll-tilt thresholds (log-transformed version) corresponded to a 5.6-fold increase in the odds of failing the balance test. We further emphasize that extrapolating the current data to fall risk has limitations. Nonetheless, the range of estimated deaths potentially due to vestibular dysfunction (Table A1 in Appendix) suggests the scope of the problem and highlights the need for broader epidemiologic studies focused on mortality associated with vestibular dysfunction.

We close this implications section by juxtaposing some facts discussed above. (1) Data showed that vestibular thresholds, including roll-tilt thresholds, broadly increased with age above the age of 40 (by 15–83% per decade depending upon the motion condition). (2) Analyses showed that balance test failures, which have previously been shown to correlate highly with falls (25), increased significantly as roll-tilt thresholds increased. (3) Calculations suggested that vestibular dysfunction could possibly be ranked somewhere between the third and tenth biggest killer of Americans. Even in isolation, this is alarming. But, given the rapid aging of the world’s population [e.g., Ref. (68)], the problem will rapidly grow much worse unless existing efforts to improve vestibular screening, vestibular diagnoses, vestibular treatments, balance treatments, fall prediction, and fall prevention are accelerated.

### Brief Summary

We measured vestibular perceptual thresholds in 105 healthy humans (54F/51M) ranging from 18 to 80 years of age. We found that thresholds significantly increased above the age of 40 for all five motion directions investigated. Even taking age and other factors into consideration, we found a significant correlation of balance test failures with increasing roll-tilt thresholds.

## Author Contributions

MB, TC, and DM designed the study and assisted in statistical analyses and interpretation and manuscript preparation. WW, YB, and TL also assisted in manuscript preparation, and statistical analyses and interpretation.

## Conflict of Interest Statement

The authors declare that the research was conducted in the absence of any commercial or financial relationships that could be construed as a potential conflict of interest.

## Footnotes

^1^Prior to multiple comparison correction, only the normalized *Z*-translation bias for subjects who passed the balance test suggested a statistically significant effect (*t*-test, *p* = 0.0441).

^2^A reviewer helpfully suggested that the paper might be better off without the appendix. After dialog engendered by the Frontier’s process, this footnote has been added to specifically note that we all agree that (a) the estimated range is large because available data do not allow solid estimates, (b) the issue was too important to gloss over by ignoring it, (c) the only way to resolve the matter is to obtain better data, and (d) including these broad estimates is likely to encourage the acquisition and analysis of better data.

## Appendix

### A. Estimating Annual Fatalities Related to Vestibular Dysfunction

According to the CDC online web-based injury statistics query and reporting (69), 26,734 persons over 40 years of age died in the US in 2011 as a direct result of unintentional falls. This is consistent with a National Vital Statistics Report (70) that stated that 26,009 persons died in the US in 2010 as a direct result of unintentional falls. Of course, only some of the 26,734 fall-related deaths were due to vestibular dysfunction. To provide this estimate of deaths related to vestibular dysfunction, some simple calculations are required. Such calculations require that we know (a) the prevalence of vestibular dysfunction and (b) the increased risk of fall due to vestibular dysfunction, both of which were discussed earlier. Specifically, we will use (a) the 35.4% estimate of prevalence provided by Agrawal et al. (25), because it is the most comprehensive broad assessment based on objective measures, and (b) the reported odds ratio of 6.3 for those individuals over the age of 40 defined as having vestibular dysfunction using the balance metric (25), because it arises from the same comprehensive data set. We note that using these estimates yield a smaller (i.e., more conservative) estimated death rate than if we used the 27% prevalence rate for vestibular dysfunction with clinical symptomology with the 12.3 odds ratio for falls in this population.

If the total pertinent population at a point in time is *x*, then 35.4% (0.354*x*) have vestibular dysfunction and 64.6% (0.646*x*) do not. Let us represent the risk of falling without vestibular dysfunction as *w*. Then, the risk of falling with vestibular dysfunction [as defined by Agrawal et al. (25)] is 6.3 times higher (6.3*w*). While it can be argued that the risk of a bad fall for those with vestibular dysfunction is likely higher than for those with normal function, we conservatively assume that the risk of death due to a fall is independent of vestibular status (*y*). The total number of US deaths directly due to falls (26734) equals the sum of deaths in those with vestibular dysfunction (0.354 × 6.3 *w x y* = 2.23*z*) and those without vestibular dysfunction (0.646 *w x y* = 0.646*z*), where *z* = *w x y*, so 26,734 = 2.23*z* + 0.646*z*. This can be solved for *z*, which equals 9296; therefore, 26,734 = 2.23(9296) + 0.646(9296); thus, we can estimate that 20,729 (2.23 × 9296) equals the number of fall deaths in those individuals with vestibular dysfunction. Of course, these individuals had a chance of falling independent of their vestibular status. By definition, this risk is the same as for individuals without vestibular dysfunction, so 3,291 (0.354 × 9296) of these would have fallen independent of their vestibular dysfunction, leaving an estimated 17,438 deaths directly attributable to falls due to vestibular dysfunction. These calculations suggest that 65% of deaths directly attributable to falls are related to vestibular dysfunction (We note that death due to falling is relatively rare; so, risk and odds here would be nearly equivalent).

But falls also indirectly lead to death. For example, falls cause hip fractures, among other injuries, and hip fracture has a high mortality rate following fracture. There were 306,000 hospital admissions for hip fractures in 2010 (71), and it has been estimated that more than 95% are due to falls (72). One study reported that the overall 1-year post-fracture mortality rate was 27.3% and further reported that mortality after hip fracture at the end of the follow-up was 79.0% (73). If we conservatively use the 1-year mortality rate of 27.3%, this suggests that more than 79,361 patients die each year following hospitalization for a hip fracture. Repeating the exact same calculations outlined in the previous paragraph, we estimate that there may be 51,767 deaths correlated with falls (i.e., following hip fracture) that are related to vestibular dysfunction. As for the calculations in the previous paragraph, some fraction of these deaths is unrelated to the fall and hip fracture. The same study reported an average death risk ratio of 3.26 in this hip fracture population relative to an age-matched population. Analogous to the above calculations, the total number of deaths equals those related to the hip fracture and those that would have occurred anyway (51,767 = 3.26*v* + *v*), yielding the estimated number of deaths related to the hip fracture as 39,615. Simply summing these two death estimate numbers (i.e., the number of fall deaths directly and indirectly related to vestibular dysfunction) yields an estimate that vestibular dysfunction contributes to 57,053 deaths each year.

Given the conservative nature of these estimates (using only 1-year hip fracture mortality, not including traffic and other non-fall accidents, using the lower odds ratio, etc.), these estimates suggest that vestibular dysfunction likely contributes to more than 57,000 deaths each year. If categorized this way, according to a national vital statistics report (74), this would rank number 10 on the list of leading causes of death in 2010 behind heart disease (598,000), cancer (575,000), chronic respiratory diseases (138,000), stroke (129,000), accidents (121,000), Alzheimer’s (83,000), and diabetes (69,000). Table A1 in Appendix provides a range of estimates – some more conservative and some less so. The annual death estimates correlated with vestibular dysfunction range from 48,000 to 152,000.

**Table A1:** **Deaths due to vestibular dysfunction under different assumptions**.

Prevalence (%)	Falling odds ratio	Deaths	Fall death due to VD (%)	Fall death due to VD	Admissions for hip fractures	Percent fractures due to falls (%)	Hip fracture mortality rate (%)	Deaths indirectly attribute to fall related to VD	Death from hip fracture, indirectly related to VD	Total estimated deaths
35.4	6.3	26,734	65.23	17,438	306,000	95	27.3	79,361	39,615	57,053
27	12.3	26,734	75.31	20,133	306,000	95	27.3	79,361	45,732	65,865
22.9	6.3	26,734	54.83	14,657	306,000	95	27.3	79,361	33,297	47,954
35.4	6.3	26,734	65.23	17,361	306,000	95	79	229,653	114,637	131,998
27	12.3	26,734	75.31	20,133	306,000	95	79	229,653	132,090	152,223
22.9	6.3	26,734	54.83	14,657	306,000	95	79	229,653	96,355	111,012

*The top row shows the values described in detail in the text. The second row changes only the prevalence for vestibular function accompanied by clinical symptomology and the matching odds ratio from the American study (25). The third row uses the prevalence reported in the German study (65) with the most conservative odds ratio of 6.3 from the American study (25). The next three rows repeat the calculations from the first three rows but now using the hip fracture mortality rate of 79% at the end of follow-up (73)*.
